# Reply to “A commentary on ‘Self-expanding metal ureteral stent for ureteral stricture: experience of a large- scale prospective study from a high-volume center – cross-sectional study’ (Int J Surg 2021; 95: 106161)”

**DOI:** 10.1097/JS9.0000000000000895

**Published:** 2023-11-16

**Authors:** Cai Tang, Xin Wei

**Affiliations:** Department of Urology, West China Hospital of Sichuan University, Chengdu, People’s Republic of China

*Dear Editor*,

We are immensely grateful for the valuable insights provided by the team of J.S. Wang^1^. The questions raised have prompted profound reflection and have been instrumental in driving the progress of our entire research. After thorough discussion, we would like to offer the following response to this significant and uniquely insightful comment. We hope the authors will find it satisfactory.

The authors proposed four questions. In fact, questions one and three address a similar issue: the discussion of how to reduce displacement. Specifically, is it necessary to place more than two stents to solve this issue? If it is an anatomical narrowing, after placing the stent, we can use the anatomical structures to secure the stent in place. If it is a functional narrowing, we choose to place multiple stents, and there will be some overlap between adjacent stents. This provides friction, which helps to simultaneously secure the adjacent stents. Typically, three stents can connect the renal pelvis and the bladder, ensuring the entire ureter remains unobstructed. Regarding the topic of multiple stents leading to vesicoureteral reflux, if the lower urinary tract is unobstructed, persistent hydronephrosis will never occur. The last question also carries significant weight. It concerns whether the stent generates stones. If the patient does not have a predisposition to forming kidney stones, simply drinking an adequate amount of water is sufficient to prevent stone formation^2,3^. However, if the patient is prone to kidney stones, they should drink plenty of water. Even if stones do form, they are usually small calcifications or tiny grains adhering to the inner surface of the stent. Regular follow-up examinations should be conducted, and if stone formation is detected, the stent should be replaced. Currently, the reported effective lifespan of a stent is 1–3 years^3,4^. Annual radiological follow-up and regular testing of creatinine levels and urine are indicators to determine whether a stent replacement is necessary. If there are no abnormalities, we typically replace the stent every 3 years. As mentioned earlier, if there is stone formation or a severe urinary tract infection, early replacement may be required^4^.

In summary, we are deeply appreciative of these constructive comments and hope that the authors can continue to provide valuable insights to assist us in making further advancements. We aim to meet the author’s expectations with our response.

## Ethical approval

No applied.

## Consent

No applied.

## Sources of funding

No applied.

## Author contribution

C.T.: wrote the paper; X.W.: came up with the idea.

## Conflicts of interest disclosure

There are no conflicts of interest.

## Research registration unique identifying number (UIN)

No applied.

## Guarantor

No applied.

## Data availability statement

No applied.
